# Dynamic methylation and expression of alternative promoters for oestrogen receptor alpha in cell line models of fulvestrant resistance

**DOI:** 10.1002/1878-0261.13713

**Published:** 2024-08-06

**Authors:** Juliane Albrecht, Mirjam Müller, Völundur Hafstað, Kamila Kaminska, Johan Vallon‐Christersson, Gabriella Honeth, Helena Persson

**Affiliations:** ^1^ Department of Clinical Sciences Lund, Oncology Lund University Cancer Centre Sweden

**Keywords:** breast cancer, drug resistance, fulvestrant, methylation, oestrogen receptor, promoter

## Abstract

Oestrogen receptor alpha (ER; gene symbol *ESR1*) is the most important prognostic and treatment‐predictive biomarker in breast cancer. Drugs targeting oestrogen and ER for endocrine therapy of breast cancer include aromatase inhibitors, the selective ER modulator tamoxifen and the selective ER degrader fulvestrant. Tumours can develop resistance to endocrine therapy through several mechanisms, which is often linked to altered expression of ER. To investigate the role of promoter methylation in the regulation of *ESR1* expression, we used bisulfite sequencing to measure methylation at CpG sites in alternative ER promoter regions for six cell line models of fulvestrant resistance. Both CpG methylation and expression of alternative first exons changed dynamically, with striking differences between cell lines that had stable or unstable resistance upon fulvestrant withdrawal. Methylation at some CpG sites was strongly negatively correlated with expression of specific first exons. In a breast tumour cohort, higher relative expression of upstream alternative first exons was associated with worse prognosis in post‐menopausal women with ER‐positive tumours who received endocrine therapy.

## Graphical abstract

Oestrogen receptor alpha (ER) is a prognostic biomarker and target for endocrine therapy in breast cancer. Resistance to the ER‐targeting drug fulvestrant is associated with altered ER expression. Bisulfite sequencing of fulvestrant‐resistant cell lines revealed dynamic promoter methylation and first exon expression, correlating with resistance stability. In post‐menopausal women, higher upstream exon expression predicted a worse prognosis after endocrine therapy.

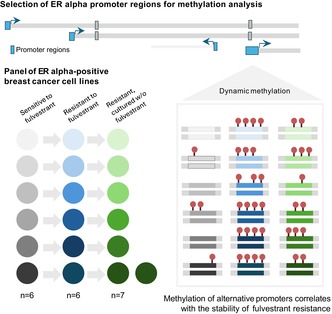

**Abbreviations**
AI: aromatase inhibitor
ATCC: American Type Culture Collection
CDK: cyclin‐dependent kinase
CI95: 95% confidence interval
DRFI: distant recurrence‐free interval
DSMZ: German Collection of Microorganisms and Cell Cultures GmbH
ER: oestrogen receptor alpha
fpkm: fragments per kilobase of exon model and million reads
FR: fulvestrant‐resistant subline
FR‐F: fulvestrant‐resistant subline cultured without fulvestrant
lncRNA: long non‐coding RNA
LTED: long‐term oestrogen deprivation
OS: overall survival
P: parental cell line
PCR: polymerase chain reaction
PR: progesterone receptor
RFI: recurrence‐free interval
RIPA: radioimmunoprecipitation assay buffer
RNA‐Seq: RNA sequencing
RT‐PCR: reverse transcription polymerase chain reaction
SCAN‐B: The Sweden Cancerome Analysis Network – Breast
SD: standard deviation
SERD: selective oestrogen receptor degrader
SERM: selective oestrogen receptor modulator
STR: short tandem repeat
TCGA: The Cancer Genome Atlas
T‐DMR: tissue‐dependent and differentially methylated region
TFBS: transcription factor binding site
uORF: upstream open reading frame

## 1 Introduction

Oestrogen receptor alpha (ER, official gene symbol *ESR1*) is a ligand‐activated transcription factor that belongs to the nuclear receptor superfamily. It is expressed in the mammary glands and female reproductive tract, as well as in liver, muscle, adipose tissue, and the pituitary gland [1]. Approximately 75% of breast cancers express the ER, which contributes to tumour development and progression [2]. ER expression is an important biomarker in clinical management of breast cancer, where it is used as a positive prognostic and treatment‐predictive factor. ER‐positive breast cancers are generally responsive to endocrine therapies, including those targeting oestrogen synthesis (aromatase inhibitors, AI), selective oestrogen receptor modulators (SERMs) like tamoxifen, and selective oestrogen receptor degraders (SERDs) like fulvestrant [3]. During prolonged treatment, tumours can acquire resistance to endocrine therapy [4]. This often coincides with changes in ER status and is associated with more aggressive tumour behaviour and poor prognosis [5, 6]. For patients failing tamoxifen therapy, treatment with fulvestrant can provide benefits due to its unique mechanism of action. Fulvestrant is classified as a ‘pure’ antagonist lacking oestrogenic activity and binds to the receptor to prevent dimerization, leading to destabilisation and rapid degradation of the ER‐fulvestrant complex and loss of oestrogen signalling [7].

The mechanisms underlying resistance to endocrine therapy are complex and include e.g. mutations of the ER, kinase activation and phosphorylation of the ER to promote ligand‐independent signalling, epigenetic silencing of ER expression and activation of growth factor receptors including EGFR and ERBB2 [8, 9, 10]. Epigenetic mechanisms that alter gene expression include DNA methylation of cytosines in CpG dinucleotides, typically in proximal promoter regions [11]. Several breast cancer cell line models have been developed to study resistance to endocrine therapy *in vitro*. These include both studies where cells are cultured in increasing concentrations of the drug of interest and studies where cells are subjected to long‐term oestrogen deprivation (LTED) to mimic the effect of aromatase inhibition [12, 13]. The MCF7 breast cancer cell line is a commonly used model where tamoxifen can even stimulate growth in resistant cells [14]. Altered growth factor receptor signalling can also be involved in resistance to tamoxifen [15]. Tamoxifen‐resistant cells typically retain expression of the ER and respond to second‐line therapies such as fulvestrant [14].

Fulvestrant‐resistant MCF7 and T‐47D breast cancer cells can have downregulation of the ER with upregulation of ERBB2 and hormone‐independent growth [16]. We have previously developed and characterised six breast cancer cell line models with resistance to fulvestrant [17]. The cell lines adapted at different points in the cell cycle to promote progression and had distinct responses to cyclin‐dependent kinase (CDK) inhibitors. Overexpression of cyclin E2 was seen in cells with stable fulvestrant resistance and higher expression was associated with shorter progression‐free survival in patients with metastatic disease who received fulvestrant.

Changes in methylation of CpG sites in promoter regions with concomitant deregulation of ER expression has been detected in MCF7 cells subjected to LTED as well as in MCF7 and T47D cells resistant to fulvestrant [18, 19]. Acquisition of resistance to tamoxifen has also been associated with altered methylation of ER target genes while methylation leading to loss of ER signalling and activation of compensatory growth‐stimulatory pathways was seen in resistance to fulvestrant [20].

Here we have investigated the role of ER promoter methylation in a panel of six different ER‐positive breast cancer cell lines with matched fulvestrant‐resistant sublines and resistant sublines cultured in the absence of fulvestrant. The characteristics of these cells vary and include dynamic changes in ER expression and resistance that ranges from rapid reversion to long‐term stability [17]. We found strong negative correlation between methylation at specific sites and use of alternative ER promoters, as well as changes in methylation patterns that are consistent with altered ER expression in the different sublines. We also found that a proportionally higher expression of upstream promoters is associated with worse prognosis in post‐menopausal women with ER‐positive breast cancer who receive endocrine therapy. Lastly, we identified a region in an *ESR1* intron that has three binding sites for the transcriptional insulator CCCTC‐binding factor (CTCF) where CpG methylation is strongly negatively correlated with ER expression in both tumours and cell lines. This region could potentially act as a master regulator of ER status through chromatin remodelling.

## 2 Materials and methods

### 2.1 Cell culture

The oestrogen receptor‐positive breast cancer cell lines CAMA‐1 (RRID:CVCL_1115), HCC1428 (RRID:CVCL_1252), MCF7 (RRID:CVCL_0031), T‐47D (RRID:CVCL_0553), and ZR‐75‐1 (RRID: CVCL_0588) were obtained from American Type Culture Collection (ATCC, Manassas, VA, USA) and EFM‐19 (RRID:CVCL_0253) was obtained from German Collection of Microorganisms and Cell Cultures GmbH (DSMZ). CAMA‐1 and MCF7 cell lines were cultured in DMEM/F12 (Cytiva/HyClone, Marlborough, MA, USA), and EFM‐19, HCC1428, T47D, and ZR‐75‐1 cell lines in RPMI‐1640 (Cytiva/HyClone). All culture media were supplemented with 10% fetal bovine serum (Cytiva/HyClone) and cells were cultured at 37 °C, 5% CO_2_ in a humidified atmosphere. Cells were confirmed to be mycoplasma‐free with the MycoAlert PLUS Mycoplasma Detection Kit (Lonza, Basel, Switzerland). Development of the fulvestrant‐resistant sublines by exposure of parental cells to increasing concentrations of fulvestrant from 100 pm to 100 nm has been described in [17]. For maintenance, fulvestrant‐resistant (FR) sublines were grown in complete medium supplemented with 100 nm fulvestrant. Cell line identity was authenticated by short tandem repeat (STR) profiling (Eurofins Genomics, Luxembourg, Luxembourg).

### 2.2 Western blotting

Cells were lysed in RIPA buffer (1% Triton X‐1000, 1% sodium deoxycholate, 10 mm Tris–HCl, pH 8.0, 1 mm EDTA, 0.5 mm EGTA, 0.1% SDS, 140 mm NaCl; all Sigma‐Aldrich/Merck, Burlington, MA, USA) supplemented with cOmplete, Mini, EDTA‐free Protease Inhibitor Cocktail (Roche/Merck, Basel, Switzerland) for western blotting. Protein concentrations were determined using the Pierce BCA Protein Assay Kit (Thermo Fisher Scientific, Waltham, MA, USA). For SDS/PAGE, 7.5 μg of protein per sample was loaded on a 4–20% Mini‐PROTEAN TGX Stain‐Free gel with Precision Plus Protein All Blue Prestained Protein Standards as molecular weight marker and Trans‐Blot Turbo Mini 0.2 μm PVDF Transfer Packs were used in blotting (all from Bio‐Rad, Hercules, CA, USA). The C‐terminal anti‐ER antibody (sc‐543; Santa Cruz Biotechnology, Dallas, TX, USA) was diluted 1 : 500 and the anti‐α‐tubulin antibody (ab7291; abcam, Cambridge, UK) 1 : 5000. Secondary antibodies were HRP‐conjugated goat anti‐rabbit (31460; Invitrogen/Thermo Fisher Scientific, Carlsbad, CA, USA) and anti‐mouse (31430; Invitrogen/Thermo Fisher Scientific), both diluted 1 : 10 000. All antibodies were diluted in 5% milk‐TBST. Signals were detected using Clarity Western ECL Substrate (Bio‐Rad) and imaged on a ChemiDoc MP Imaging system (Bio‐Rad).

### 2.3 DNA extraction, bisulfite conversion and PCR

DNA extraction was performed with the DNeasy Blood & Tissue kit (Qiagen, Hilden, Germany) for 4 million cells per sample. DNA concentrations were determined using a NanoDrop 8000 Spectrophotometer (Thermo Fisher Scientific). The EZ DNA Methylation‐Lighting Kit (Zymo Research, Irvine, CA, USA) was used for bisulfite conversion of 1 μg of genomic DNA per sample. The fragment length distribution for converted DNA was assessed for a subset of 10 samples on a 2100 Bioanalyzer using the RNA 6000 Pico kit (both from Agilent, Santa Clara, CA, USA). The RNA assay was used since it was expected to be more suitable for single‐stranded DNA. The size distribution is shown in Fig. S1. Primers for amplification of bisulfite‐converted DNA are included in Table S1. Twelve regions of 300–450 bp were selected based on proximity to annotated transcription start sites and CpG site clustering. PCR amplification was done using KAPA HiFi HotStart Uracil ReadyMix Kit (Roche) with the following conditions: 95 °C for 3 min, followed by 35 cycles of 98 °C for 20 s, 55–60 °C for 15 s, 72 °C for 30 s, and a final extension step of 72 °C for 1 min. The annealing temperature was adjusted to the specific primer pairs, as shown in Table S1. Each reaction contained 300 nm final concentration each of forward and reverse primers, 1× KAPA HiFi HotStart Uracil ReadyMix, 0.5 m betaine, 75 ng bisulfite‐converted DNA, and nuclease‐free water to a total volume of 25 μL. Amplification was verified using acrylamide gel electrophoresis.

### 2.4 Sequencing library preparation and next‐generation sequencing

The 12 amplicons were pooled per sample, resulting in 19 amplicon pools which were purified using the HighPrep PCR Clean‐up System (MagBio Genomics Inc., Gaithersburg, MD, USA) at a 1 : 1 ratio to remove fragments below 250 bp. The DNA concentration was determined using the Qubit dsDNA Broad Range Assay Kit (Invitrogen). The NEBNext Ultra II DNA Library Prep with beads and NEBNext Multiplex Oligos for Illumina (both from New England Biolabs, Ipswich, MA, USA) were used for preparation of sequencing libraries from 400 ng pooled and purified PCR products per sample. Library quality was evaluated using a Bioanalyzer with a High Sensitivity DNA chip (Agilent Technologies) and library concentration was determined with the Qubit dsDNA Broad Range Assay kit. Sequencing was performed using the 500‐cycle MiSeq Reagent Kit v2 on a MiSeq System (both from Illumina, San Diego, CA, USA). cutadapt v4.4 was used to trim adapters and low‐quality read ends for fastq files before methylation analysis with bismark v0.24.1. bowtie2 and ucsc hg38 were used for read alignment.

### 2.5 Pyrosequencing

Three amplicons (regions 10–12) and two sets of cell lines (HCC1428 and ZR‐75‐1) were selected for validation of CpG methylation by pyrosequencing in technical duplicates for the same bisulfite‐converted DNA. PCR was performed as before but with 5′ biotinylated reverse primers. Sequencing was performed with the PyroMark Q48 Advanced reagents kit on a PyroMark Q48 Autoprep sequencer (both from Qiagen). Sequencing primers are included in Table S1.

### 2.6 RNA extraction and real‐time RT‐PCR

Total RNA was extracted from all cell lines using TRI Reagent (Sigma‐Aldrich) and concentrations were determined with a NanoDrop 8000 Spectrophotometer. For cDNA synthesis, 400 ng of DNase‐treated RNA was used with RevertAid H Minus reverse transcriptase (Thermo Fisher Scientific) and anchored oligo(dT) primers (dT_20_VN). Primer sequences for real‐time reverse transcription polymerase chain reaction (real‐time RT‐PCR) on the CFX96 real‐time PCR detection system with iTaq Universal SYBR Green Supermix (Bio‐Rad) are included in Table S1. The expression of each first exon was normalised to the expression of *ACTB* and multiplied by 10 000 for promA to promF or by 100 for the 3′ UTR to facilitate plotting.

### 2.7 Patient data and breast tumour RNA‐Seq data analysis

The SCAN‐B study [21, 22] was conducted in accordance with the Declaration of Helsinki and has been approved by the Regional Ethical Review Board of Lund (2007/155, 2009/658, 2009/659, 2014/8), the county governmental biobank centre, and the Swedish Data Inspection group (364‐2010). Written information was given by trained health professionals and all patients provided written informed consent. Patients undergoing surgery were recruited between September 1, 2010, and March 31, 2015, from hospitals participating in SCAN‐B in Lund, Malmö, Helsingborg, Kristianstad, Växjö, Halmstad, Uppsala, Karlskrona, Varberg, and Ljungby. Library preparation and sequencing for RNA‐Seq has been described in [22]. The SCAN‐B samples included here were collected and are a subset of the cohort described in [23]. For calculation of relative first exon expression, we calculated the fraction of reads per first exon out of the total number of reads in first exons of the ER. For analysis of breast tumours (TCGA BRCA) from The Cancer Genome Atlas (TCGA) [24], Spearman's rank correlation tests were performed for 1095 samples between *ESR1* expression from RNA‐Seq data and methylation levels of CpG sites located within the *ESR1* locus (450k array probes spanning the region chr6:151600000–152000000). A correlation test was performed for each CpG site for a total of 64 tests. Final *P*‐values were corrected for multiple testing using Benjamini‐Hochberg correction. All data were obtained with the Genomic Data Commons (GDC) Data Transfer Tool Client.

### 2.8 Analysis of transcription factor binding sites and evolutionary conservation

The UCSC Table Browser [25] was used to obtain transcription factor binding sites from the UniBind robust set of transcription factor–DNA interactions [26] and phyloP scores [27]. To generate the background set for comparison of CpG site conservation, we selected protein‐coding genes with mean expression within the interquartile range of the ER in the SCAN‐B breast tumour cohort (14.5–114 fpkm, *n* = 3833 genes). The coordinates of all first exons for these genes were padded with ±750 bp and the resulting regions were masked to remove any overlapping protein‐coding sequence. Only regions longer than 400 bp containing at least four CpG sites were retained.

### 2.9 Statistical analysis

Statistical analyses and plotting were done in r version 4.3.1 (Vienna, Austria) and Microsoft Excel. Survival analysis was performed in r using the survival and survminer packages.

## 3 Results

### 3.1 Different characteristics of breast cancer cell line models of fulvestrant resistance

**Fig. 1:**
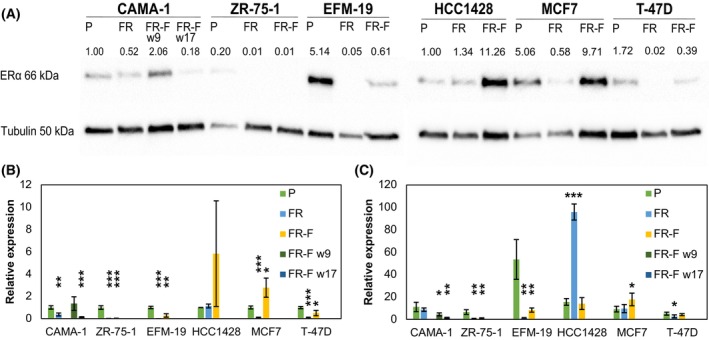
Expression of the oestrogen receptor (ER) in parental and derived sublines. Protein expression determined by immunoblotting (A). Band intensities were normalised to total protein content per lane and expressed relative to CAMA‐1 (blot 1, left) or HCC1428 parental cells (blot 2, right). In the publication by Kaminska et al. [17], FR‐F at week 9 had higher ER expression than FR but lower than the parental cell line. Membranes were reprobed for tubulin as a loading control. ER protein expression in FR and FR‐F sublines relative to parental cells (B), *n* = 3. ER mRNA expression in parental and derived sublines by real‐time reverse transcription polymerase chain reaction (real‐time RT‐PCR), *n* = 4 (C). Expression of the 3′ untranslated region (3′ UTR) was normalised to the expression of *ACTB* and multiplied by 100 to facilitate visualisation. Error bars represent standard deviation (SD), *P < 0.05, **P < 0.01, ***P < 0.001 (Student's *t*‐test). FR, fulvestrant‐resistant; FR‐F, FR cultured without fulvestrant; kDa, kilo Dalton; P, parental.

We have previously generated fulvestrant‐resistant sublines of six different ER‐positive breast cancer cell lines by culturing them in increasing concentrations of fulvestrant [17]. The resulting sublines differed in their properties including response to fulvestrant, oestradiol, and 4‐hydroxytamoxifen, the active metabolite of the SERM tamoxifen (Table 1). One of the resistant cell lines showed increased expression of the ER (HCC1428) while the remaining five had decreased expression (Fig. 1 and Figs S2–S4). Differences in gene expression profiles and response to cyclin‐dependent kinase inhibitors suggested that the resistant cells had adapted in different ways to loss of ER signalling. When fulvestrant was removed from the cell culture medium it also became apparent that the cell lines differed in the stability of resistance. Two cell lines remained resistant upon long‐term (9 weeks) withdrawal of the drug (CAMA‐1 and ZR‐75‐1), two cell lines had moderately stable resistance and reverted after 3–6 weeks (EFM‐19 and HCC1428), and two cell lines reverted to being sensitive after 1 week (MCF7 and T‐47D). Loss of resistance was always associated with re‐expression of the ER.

**Table 1:** Properties of the parental and derived sublines. 4OHT, 4‐hydroxytamoxifen; E2, estradiol; FR, fulvestrant‐resistant; FR‐F, FR cultured without fulvestrant for the indicated number of weeks; IC50, half maximal inhibitory concentration; n.d., not determined; P, parental.

Cell line	Subline	Weeks to resistance^a^	Response to fulvestrant (IC50)^a^	Proliferation stimulated in 1 nm E2^a^	Growth inhibition in 100 nm 4OHT^a^
CAMA‐1	P		Sensitive (21 pm)	Yes	Yes
	FR	11	Resistant (4.9 μm)	No	No
	FR‐F w9		Limited sensitivity (n.d.)		
	FR‐F w17		Resistant (n.d.)		
EFM‐19	P		Sensitive (183 pm)	Yes	Yes
	FR	14	Resistant (2.3 μm)	No	No
	FR‐F w11		Sensitive (n.d.)		
HCC1428	P		Sensitive (2.2 nm)	Yes	Yes
	FR	25	Resistant (9.6 nm)	No	Yes
	FR‐F w10.5		Sensitive (n.d.)		
MCF7	P		Sensitive (351 pm)	Yes	Yes
	FR	28	Resistant (2.9 μm)	Yes	Yes
	FR‐F w7		Sensitive (n.d.)		
T‐47D	P		Sensitive (0.3 pm)	Yes	Yes
	FR	40	Resistant (6.7 μm)	No	No
	FR‐F w11		Sensitive (n.d.)		
ZR‐75‐1	P		Sensitive (212 pm)	Yes	Yes
	FR	9	Resistant (6.2 μm)	No	No
	FR‐F w11		Resistant (n.d.)		

a Results from Kaminska et al. [17].

### 3.2 Dynamic changes in promoter methylation patterns

**Fig. 2:**
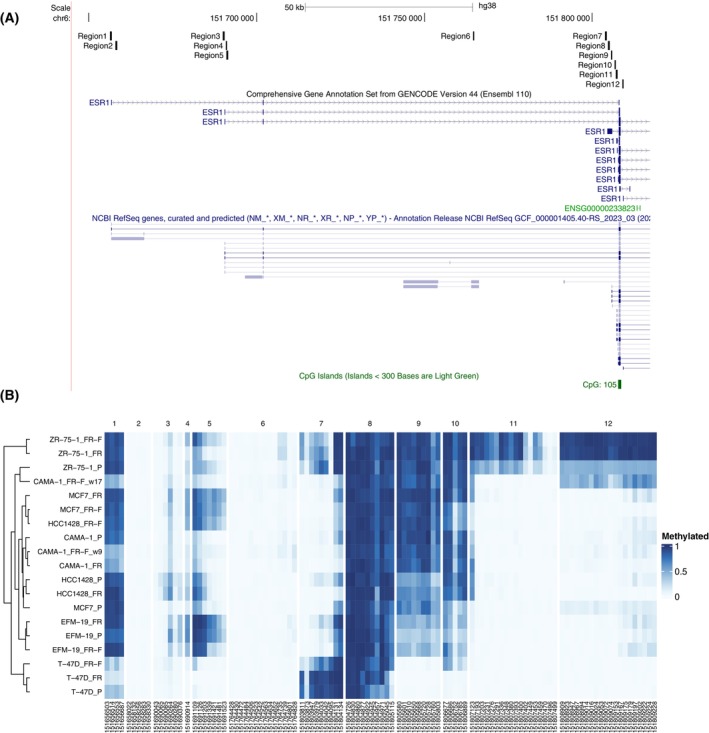
Analysis of CpG site methylation. Schematic view of the 12 analysed regions with CpG sites together with the different first exons of the *ESR1* locus present in GENCODE and RefSeq transcript annotation (A). Heatmap showing the fraction of methylated reads per CpG site and sample with regions 1–12 marked (B). FR, fulvestrant‐resistant; FR‐F, FR cultured without fulvestrant; P, parental.

**Fig. 3:**
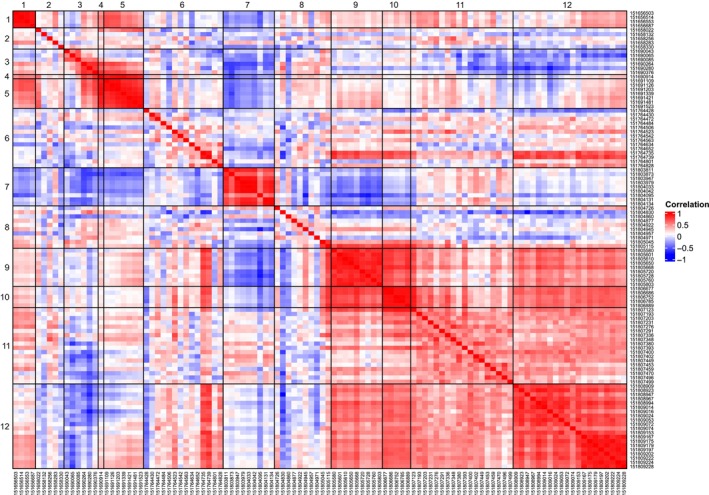
Heatmap showing Spearman rank correlation between CpG sites for the fraction of methylated reads across all samples. Regions 1–12 are labelled.

The striking differences between cell lines and the dynamic changes in ER expression and drug sensitivity over the course of weeks led us to hypothesise that an epigenetic mechanism such as promoter methylation might be involved. We therefore identified clusters of CpG sites located near alternative promoter regions within the ESR1 locus based on GENCODE and RefSeq transcript annotation. We then amplified 12 approximately 400 bp regions from bisulfite‐converted DNA for 19 sublines including parental (P) and resistant (FR) cells, as well as resistant cells cultured without fulvestrant (FR‐F) for the six cell lines (two time points were included for CAMA‐1 FR‐F where increased sensitivity to fulvestrant was not seen until 9 weeks after withdrawal of the drug). Eleven amplicons were associated with six different first exons and one with the first exon of the intronic antisense long non‐coding (lnc) RNA LOC107986529 (Fig. 2A). Amplicons were pooled and barcoded by sample before sequencing on an Illumina MiSeq and the fraction of methylated reads was calculated for 108 CpG sites (Table S2). Sequence data statistics are included in Table S3. As shown in Fig. 2B, there were striking differences in promoter methylation among the different cell lines, although the parental, FR and FR‐F sublines often cluster together. The ZR‐75‐1 FR‐F and CAMA‐1 FR‐F w17 sublines, which were characterised by long‐term stable resistance and downregulation of ER expression in the absence of fulvestrant, have high methylation of regions 11 and 12 that are located just upstream and downstream, respectively, of the main promoter region and the first coding exon. These regions are almost completely demethylated in all other sublines. When comparing methylation levels across all sublines, CpG sites located within the same or nearby regions are typically closely correlated while sites near different alternative promoters often have low or even negative correlation (Fig. 3).

**Fig. 4:**
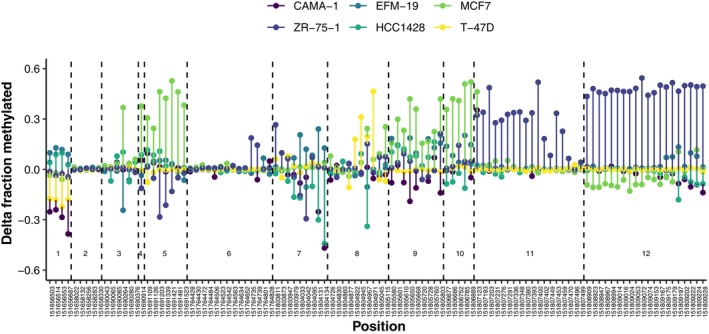
Differences in the fraction of methylated reads at CpG sites between fulvestrant‐resistant sublines and their respective parental cell line. The methylated fraction in the parental cell line was subtracted from the methylated fraction in the resistant subline so that positive values correspond to increased methylation and negative values to decreased methylation in resistant cells. Regions 1–12 are labelled.

A comparison across cell lines of the changes in methylation patterns between FR and parental sublines highlighted both differences and similarities in adaptation to growth in fulvestrant (Fig. 4). Although five of six FR sublines have decreased ER expression, this is associated with increased methylation in regions near different alternative promoters. For example, the resistance of MCF7 and T‐47D FR cells was highly unstable and both sublines reverted to a sensitive, ER‐expressing state within a week. However, the changes in promoter methylation between parental and FR sublines are clearly different with relatively few alterations in T‐47D, many in MCF7, and very little overlap between them. HCC1428 FR, the only resistant subline with increased expression, has moderately decreased methylation in several regions.

**Fig. 5:**
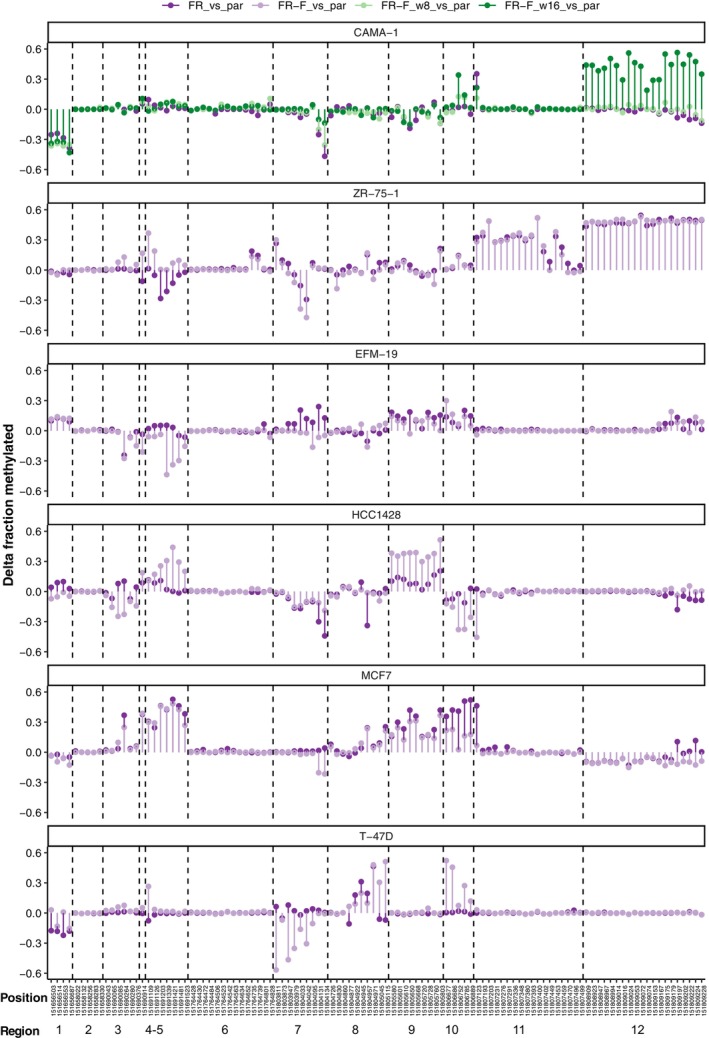
Differences in the fraction of methylated reads at CpG sites between fulvestrant‐resistant sublines, fulvestrant‐resistant sublines cultured without fulvestrant, and their respective parental cell line. The methylated fraction in the parental cell line was subtracted from the methylated fractions in the sublines so that positive values correspond to increased methylation and negative values to decreased methylation compared to parental cells. Regions 1–12 are labelled. FR, fulvestrant‐resistant; FR‐F, FR cultured without fulvestrant; P, parental.

Changes in promoter methylation between FR and FR‐F cells compared to the parental cell lines are contrasted in Fig. 5. It is striking that CAMA‐1 FR only has a single CpG in region 11 with clearly increased methylation while the FR‐F subline, which maintains resistance even after 17 weeks in culture without fulvestrant, has developed near‐complete methylation of CpG sites in region 12. In ZR‐75‐1, both the FR and FR‐F sublines show similar and high methylation of regions 11 and 12. Long‐term resistance and silencing of ER expression is therefore associated with methylation of region 12, located downstream of the first coding exon. EFM‐19 FR cells had moderately stable resistance and relatively modest increases in promoter methylation. Re‐expression of the ER in the FR‐F subline is matched by loss of methylation at several of these sites. The HCC1428 FR cells were unique among the resistant sublines in having increased ER expression. The changes in methylation pattern include both CpG sites with decreased and increased methylation where some alterations are lost and others become more pronounced in the FR‐F cell line. The rapid re‐expression of the ER that was seen in the MCF7 and T47‐D FR‐F sublines is accompanied by both loss of methylation that was gained in the FR state and by demethylation of additional regions (mainly regions 12 and 7, respectively). Although these two sublines reverted to fulvestrant‐sensitivity very quickly, they are also not identical to the parental cells.

To evaluate the accuracy of these results, we selected 22 CpG sites in regions 10–12 for validation by pyrosequencing. P, FR and FR‐F sublines were included for HCC1428 and ZR‐75‐1 and assayed in technical duplicates. Results for regions 10 and 11 are shown in Fig. S5 and for region 12 in Fig. S6. Unfortunately, sequencing failed for region 11 in HCC1428, but in general these results confirm the differences seen in Illumina sequencing for regions 10 and 11. This is also mostly true for region 12 but we note that there are discrepancies for HCC1428 with a higher estimated methylated fraction and larger differences between P and FR‐F in pyrosequencing.

A comparison with methylation in matched regions for three published microarray datasets with resistant MCF7 cells is included in Fig. S7. One of these compared the parental MCF7 cell line with sublines resistant to fulvestrant, tamoxifen, and LTED [28]. Expression of the ER was absent in the fulvestrant‐resistant cells, unchanged or slightly downregulated in tamoxifen‐resistant cells, and moderately increased in LTED cells [29]. Fulvestrant resistance was associated with increased methylation of CpG sites in regions 8–12 (Fig. S7B), which is in alignment with our results. As expected from the small changes in ER expression, the tamoxifen‐resistant cells had moderately increased methylation of the same regions while cells subjected to LTED only had minimal differences. The same tamoxifen‐resistant subline has been analysed in a second study [30] with similar results (Fig. S7C). In a third study [31], another tamoxifen‐resistant MCF7 subline had increased methylation in regions 9–10 (Fig. S7D), similar to our fulvestrant‐resistant MCF7 cells. Unfortunately, there is no information on ER expression.

By analysing methylation with sequencing instead of e.g. microarrays we also had the possibility to study methylation patterns across reads. Since each read corresponds to one original DNA strand, this allows us to study the combined methylation status for all CpG sites in a region. Frequencies of the most common methylation patterns per region and cell line are shown in Figs S8–S10. The parental cell lines display strikingly heterogeneous methylation patterns for most regions, demonstrating the existence of several subpopulations. The frequencies of individual patterns differ between parental, FR, and FR‐F sublines, but in most cases patterns that are common in the sublines exist already in the parental cell line. This is compatible with a model where different subpopulations of cells expand or contract in response to selective pressure, although we of course cannot exclude the possibility that the observed changes instead depend on active methylation or demethylation. In a few cases this is the most likely explanation, see e.g. hypermethylation of region 11 in ZR‐75‐1 and region 12 in CAMA‐1 (Fig. S10).

### 3.3 Functional annotation of CpG sites

Methylation of one CpG site in our region 9 has previously been shown to interfere with binding of the ETS2 transcription factor and ER expression was negatively correlated with methylation at this site in ER‐positive tumours [19]. We analysed the overlap between our 108 CpG sites and transcription factor binding sites (TFBSs) from the UniBind 2021 robust set of direct transcription factor–DNA interactions [26]. At least one overlapping TFBS was found for 41 CpG sites (Table S2). Binding sites for several transcription factors that have previously been indicated in ER biology were found in the experiments performed in breast cell lines. These included GATA3, JUND, MYC, NR3C1 (the glucocorticoid receptor), RUNX1/2, and TFAP2C, as well as the ER itself. Additional studies have validated promoter binding and regulation of ER expression by both GATA3 and TFAP2C [32, 33]. The UniBind data also included binding sites for HOXB3 and WT1 that have been previously confirmed to regulate expression of the ER [34, 35].

**Fig. 6:**
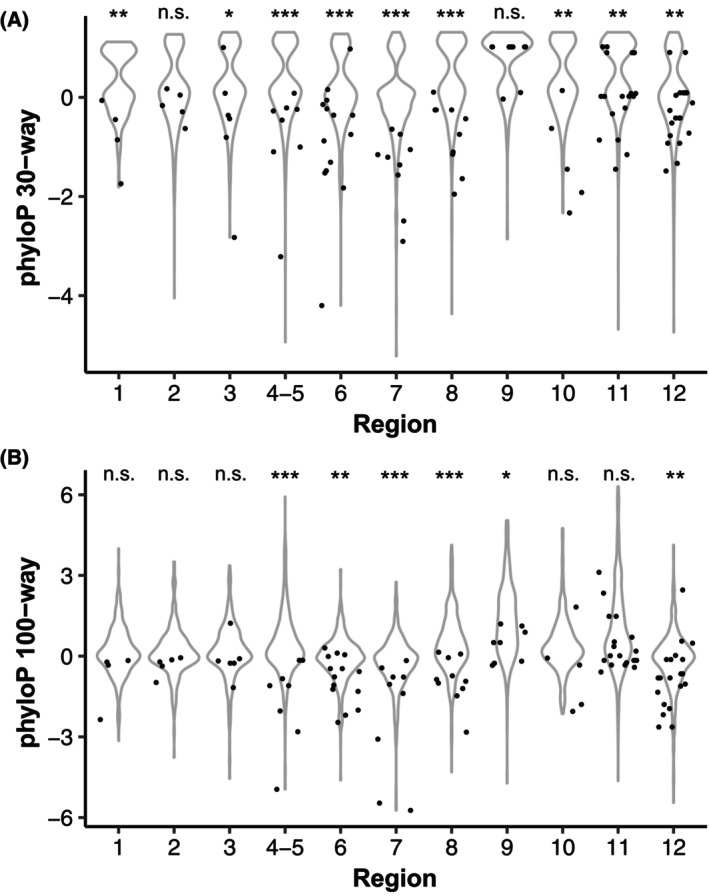
Evolutionary conservation analysis of CpG sites. Conservation scores from phyloP for mammals (A, 30‐way multiple sequence alignment) and vertebrates (B, 100‐way multiple sequence alignment). The distribution of conservation scores is shown per region as violin plots with C nucleotides in CpG sites overlaid as data points. Scores from phyloP are defined as −log_10_ of the *P*‐value under a null hypothesis of neutral evolution, where positive values correspond to conservation and negative values indicate accelerated evolution. *P < 0.05, **P < 0.01, ***P < 0.001; n.s., not significant (Wilcoxon rank sum test).

We also compared evolutionary conservation of the C nucleotides of CpG sites with surrounding nucleotides in each region using phyloP scores [27] calculated for mammals and vertebrates (Fig. 6). The absolute values of phyloP scores correspond to −log_10_ of the *P*‐value under a null hypothesis of neutral evolution. Genomic positions with neutral evolution receive scores around 0, while conserved positions have positive scores and rapidly evolving positions have negative scores. The C nucleotide positions were significantly less conserved than the surrounding sequence across all regions among both mammals (*P* < 2.2e‐16, Wilcoxon rank sum test) and vertebrates (*P* = 3.8e‐15, Wilcoxon rank sum test). Several positions show strongly negative phyloP scores, suggestive of accelerated evolution (see also Table S2). To test if this is a general property of CpG sites or specific for the ER, we generated a background set of regions surrounding transcription start sites of approximately 3800 protein‐coding genes with similar expression level in breast tumour RNA‐Seq data (see Section 2 for a detailed description). The C nucleotides in CpG sites have significantly lower phyloP scores than other positions also in the background set for both mammals and vertebrates (both *P* < 2.2e‐16, Wilcoxon rank sum test). However, CpG sites in the ER promoter regions had significantly lower phyloP scores than CpG sites in the background set (*P* = 3.5e‐6 and 3.3e‐4 for mammals and vertebrates, respectively). This effect is even more pronounced when the conservation of each C is expressed relative to the mean conservation score for the corresponding region (∆phyloP, *P* = 6.9e‐12 and 2.8e‐11 for mammals and vertebrates, respectively). The distributions of evolutionary conservation scores are shown in Fig. S11. These results show that most CpG sites in the analysed ER promoter regions are non‐conserved with lower phyloP scores than CpG sites in genes with similar expression level. Potentially, differences between species in the regulation of ER expression could explain the rapid evolution seen at some of these sites.

### 3.4 Use of alternative promoters for ER expression

**Fig. 7:**
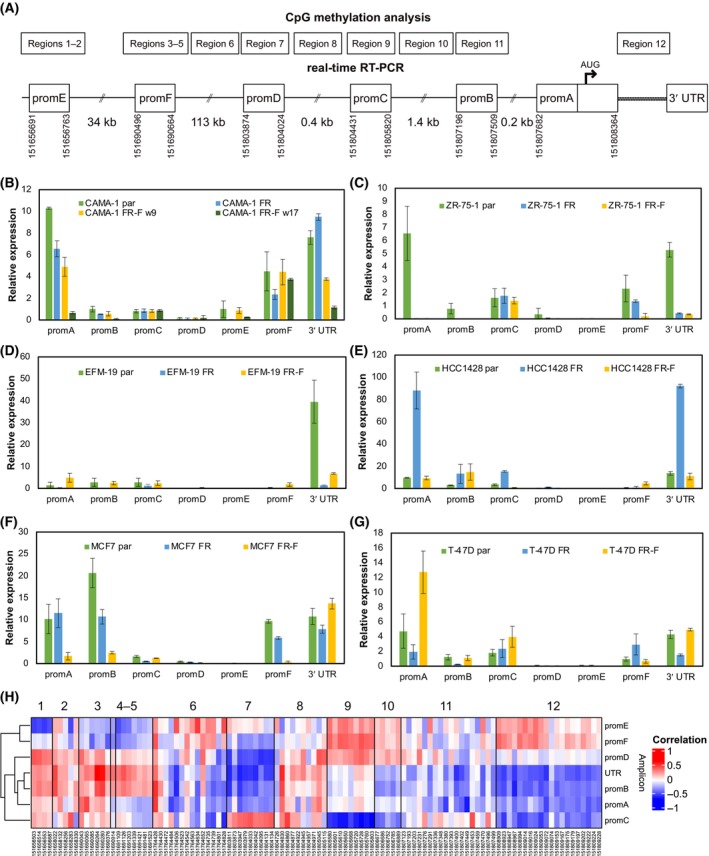
Alternative first exon expression and correlation with CpG site methylation. Schematic illustration of the 12 regions with CpG sites together with the different first exons of the *ESR1* locus that were analysed by real‐time reverse transcription polymerase chain reaction (real‐time RT‐PCR) labelled promA‐F (A). Expression of alternative first exons in parental and fulvestrant‐resistant cells (B–G). The 3′ untranslated region (3′ UTR) was included as an estimate of total oestrogen receptor (ER) expression. The expression was normalised to the expression of *ACTB* and multiplied by 10 000 for promA to promF or by 100 for the 3′ UTR to facilitate visualisation. Error bars represent standard deviation (SD), *n* = 2. Heatmap showing the Spearman rank correlation between CpG site methylation and expression of alternative promoters and the 3′ UTR across samples (H). FR, fulvestrant‐resistant; FR‐F, FR cultured without fulvestrant; P, parental.

Our analysis of CpG site methylation showed that there are large differences in methylation levels between alternative promoter regions and that sublines exhibit dynamic changes in methylation patterns where re‐expression of the ER is not necessarily a consequence of returning to the parental state. We wanted to determine how these differences were reflected in transcription of the ER and therefore measured the expression of six different alternative first exons by real‐time RT‐PCR in the 19 sublines with primers as in [36]. Location of these first exons is shown in relation to the regions used for methylation analysis in Fig. 7A. As shown in Fig. 7B–G, expression of different alternative first exons varies considerably, both among parental cells and between the different sublines for each cell line. Changes in ER expression are not associated with altered expression of all alternative first exons and some upstream promoters appear to be uncoupled from deregulation. Just as in the CpG methylation analysis, also FR‐F sublines that revert to being sensitive to fulvestrant have expression profiles that differ from the parental cells.

A comparison of promoter methylation and the expression of alternative first exons across all 19 sublines revealed interesting patterns in the correlation between methylation at specific CpG sites and isoform expression (Fig. 7H and Table S4). All first exons show a strong negative correlation between expression and methylation in nearby and overlapping regions, suggesting that CpG methylation in these regions is linked to local chromatin accessibility and transcription factor binding. The upstream‐most first exon (labelled promE) is highly negatively correlated with methylation of one CpG site in region 2. In UniBind data, this methylation site (chr6:151658283) overlaps several transcription factor binding sites found in the hepatocellular carcinoma cell line HepG2 (Table S2). The four first exons that are located close to the start of the coding sequence (promD, promC, promB, and promA) have relatively similar correlation patterns, with some notable differences. Expression of these first exons is negatively correlated with methylation of region 7, which overlaps promD, except for promC that is clearly positively correlated. The first exon promC is instead highly negatively correlated with methylation of region 9, which in turn is positively correlated with expression of the upstream first exons promE, promF and promD. In UniBind data, region 9 contains both a CTCF insulator site (found in MCF7 among other cell lines) as well as a binding site for the transcription factor aryl hydrocarbon receptor nuclear translocator (ARNT) detected in T‐47D. Correlations and the corresponding *P*‐values are included in Table S4. In total, 58 CpG sites were significantly correlated with the expression of at least one first exon across the 19 analysed samples, but none were significant after correction for multiple testing (false discovery rate).

Region 6 is not located near any of the ER promoter regions but was included in this study because of its accumulation of CpG sites and interesting location near the transcription start site of the LOC107986529 antisense lncRNA. Methylation of several CpG sites in this region is positively correlated with expression of upstream first exons (promE and promF) and negatively correlated with expression of downstream first exons (especially promC, promB, and promA). One CpG site in region 6 (chr6:151764484) overlaps a CTCF insulator element that has been detected in a multitude of samples in UniBind data and four more CTCF sites are found within a 2 kb region. The methylation levels in this region are low but, strikingly, the CAMA‐1 and ZR‐75‐1 cell lines which developed long‐term stable resistance have more methylation already in the parental cell lines (Fig. 2 and Table S2). Methylation in region 6 is also negatively correlated with total ER expression in the cell lines, as measured by expression of the common 3′ UTR (Fig. 7H). We were curious to see if this would also be the case in breast tumours and analysed matched methylation array and RNA‐Seq data for 1095 breast tumours from the TCGA BRCA cohort. Three probes were present in our region 6 and methylation was significantly negatively correlated with total ER expression for all (Table S2 and Fig. S12). We then calculated expression of LOC107986529 in breast tumour RNA‐Seq data for 3478 breast tumours from the SCAN‐B cohort [23]. Expression of the lncRNA is strongly positively correlated with ER expression as shown is Fig. S13 (Spearman's rho = 0.65, *P* < 2.20e‐16). CTCF has been shown to bind methylated CpGs with lower affinity [37] so, potentially, higher methylation is associated with decreased binding of CTCF which leads to a reorganisation of the chromatin with concomitant downregulation of ER expression from the main promoter region and increased expression from upstream promoters.

### 3.5 Alternative promoter expression in breast tumours is associated with differences in tumour characteristics and patient prognosis

**Fig. 8:**
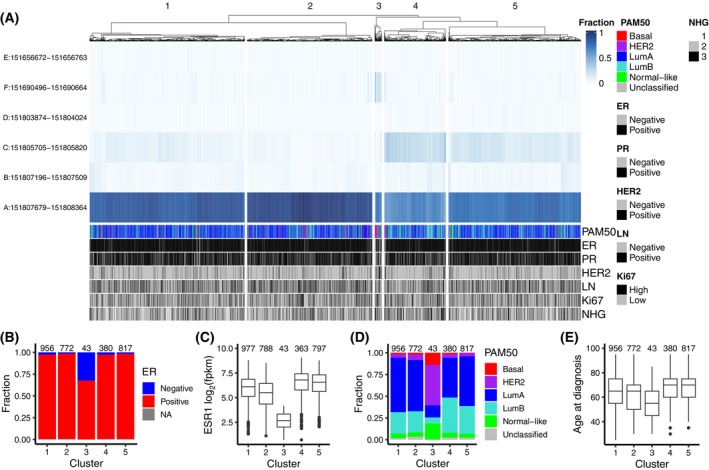
Alternative first exon expression in breast tumours. Heatmap showing the expression of alternative first exons as fraction of the total first exon expression in 2968 breast tumours (A). Annotation tracks show Prediction Analysis of Microarray 50 (PAM50) molecular subtype, Nottingham histological grade (NHG) and status for oestrogen receptor (ER), progesterone receptor (PR), the tyrosine kinase receptor ERBB2 (also known as HER2), positive lymph nodes at surgery (LN), and Ki67. ER, PR, HER2, and Ki67 status were defined by immunohistochemistry. Composition of the five indicated tumour clusters with respect to ER status (B), ER expression in log_2_ fragments per kilobase of exon model and million reads (fpkm, C), PAM50 molecular subtype (D), and patient age at diagnosis (E). The number of tumours in each cluster is indicated in the plots.

Since promoter usage and first exon expression varied among parental breast cancer cell lines and the different sublines, we wanted to explore the expression of different first exons in breast tumours. We therefore analysed expression of the different first exons in RNA‐Seq data for the SCAN‐B breast tumour cohort. When tumours were clustered by first exon expression in fragments per kilobase of exon model and million reads (fpkm), they mainly separated into clusters based on ER status (Fig. S14). To allow us to study relative expression between first exons, we therefore calculated the expression of each first exon as the fraction of the total, summed‐up first exon expression. Only the 2968 tumours that had at least 10 sequencing reads mapping to first exons were used in these calculations. As shown in Fig. 8A, the tumours now separate into several clusters where one is characterised by dominant expression of the main first exon (promA) while tumours in the other clusters also express the upstream first exons promC or promF. The composition of the different tumour clusters with respect to ER status, ER expression, molecular subtype, and patient age at diagnosis are shown in Fig. 8B–E. Relative expression of the different first exons across subgroups of tumours including ER and HER status, histological grade and molecular subtype is shown in Fig. S15.

**Fig. 9:**
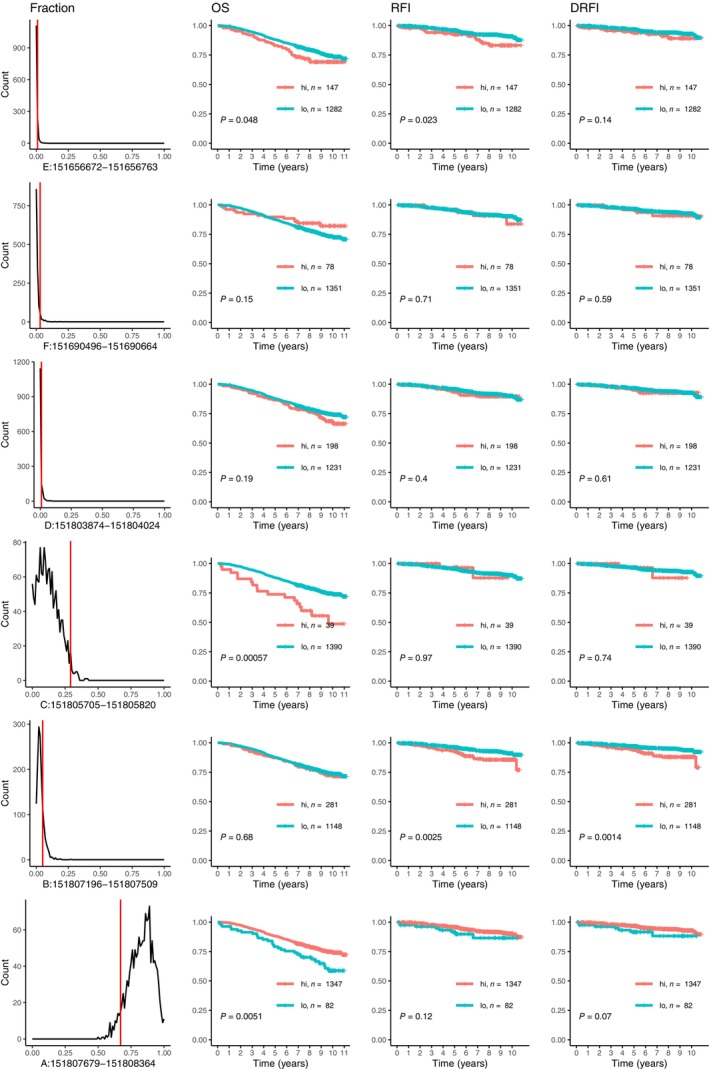
Expression of alternative first exons as fraction of the total first exon expression in 2968 breast tumours. The vertical red lines mark the cut‐offs used to define low or high expression. One first exon is shown on each row together with Kaplan–Meier curves illustrating overall survival (OS), recurrence‐free interval (RFI), and distant recurrence‐free interval (DRFI) for post‐menopausal women with oestrogen receptor (ER) positive tumours who received endocrine therapy. The log‐rank test was used to calculate *P*‐values.

We then performed a survival analysis with patients selected from the same SCAN‐B cohort for 1429 post‐menopausal women with ER‐positive breast cancer who received endocrine therapy (but not chemotherapy or anti‐HER2 therapy). As shown in Fig. 9, the analysis indicated that higher expression of the upstream first exons promE and promC in tumours is associated with shorter overall survival (OS) while higher expression of promA is associated with longer OS and recurrence‐free interval (RFI). Higher expression of promB is associated shorter RFI and distant recurrence‐free interval (DRFI), but not OS. Results of univariable and multivariable Cox regression analyses are shown in Table S5. For OS, only the association with expression of promC remained significant in a multivariable analysis with lymph node status, histological grade, and tumour size (≤ 20 mm or > 20). For RFI and DRFI, only the association with promB was significant in the multivariable analysis.

## 4 Discussion

We have analysed cytosine methylation for 108 CpG sites near alternative first exons of the ER in six different ER‐positive breast cancer cell lines with matched fulvestrant‐resistant sublines and resistant sublines cultured in the absence of fulvestrant (19 samples in total). We found that these cells display dynamic changes in ER expression with concomitant changes in promoter methylation. In general, expression of alternative first exons was anticorrelated with local CpG methylation. Two sublines that exhibited long‐term stable resistance and downregulation of the ER in the absence of fulvestrant, ZR‐75‐1 FR‐F and CAMA‐1 FR‐F w17, had high methylation near the main promoter region and the first coding exon. These regions were demethylated in all other sublines. Interestingly, sublines that reverted to a sensitive phenotype after withdrawal of fulvestrant did not return to the same methylation state as the parental cell line.

Two tissue‐dependent and differentially methylated regions (T‐DMRs) have previously been described for the ER based on bisulfite sequencing of tissues, cell lines, and breast tumours [38]. The T‐DMRs intersect with our regions 8 (near promC) and 10 (near promA and promB). The authors also identified binding sites for the transcription factor early growth response 1 (EGR1) in both regions and showed that knockdown of EGR1 decreased ER expression. Differential methylation of promoter regions was also detected in a study that used matched parental and fulvestrant‐resistant sublines of MCF7 and T‐47D [18]. Both resistant cell lines displayed downregulation of the ER, but while resistant MCF7 had increased methylation of DNA intersecting our regions 9–11 combined with stable resistance upon fulvestrant withdrawal, resistant T‐47D only had minor changes in methylation and quickly reverted in the absence of fulvestrant. A similar behaviour was observed for the T‐47D cell line also in our study. Resistance in MCF7 but not T‐47D was furthermore reported to be accompanied by activation of Akt and MAPK signalling. The same authors have also studied ER promoter methylation in oestrogen deprivation‐resistant MCF7 sublines [19]. Alternative promoter activity varied among the sublines with differential methylation around promC in DNA intersecting with our region 9. A binding site for the ETS2 transcription factor overlapping a critical CpG site was identified.

We searched the UniBind database of transcription factor binding sites collected from public ChIP‐Seq data for overlap with the analysed CpG sites. The previously reported EGR1 and ETS2 sites were not found in this database, but almost 40% of CpG sites overlapped a binding site in the UniBind database. Our analysis of evolutionary conservation showed a striking lack of conservation of CpG sites in ER promoter regions, with strongly negative phyloP scores that could suggest accelerated evolution. The difference in conservation scores was more pronounced for the ER than for the control gene set. Although the ER is evolutionarily very old [39], ER expression and function differs significantly between species [40] and it seems likely that this is regulated in part through differential methylation and alternative promoter usage.

When we analysed expression of alternative first exons in a large breast cancer cohort, tumours formed distinct clusters that were mainly defined by differences in relative expression of promA, promC, and promF. The composition of these clusters differed with respect to tumour and patient characteristics, where expression of promC was more common in tumours of the luminal B subtype and expression of promF was seen in tumours from non‐luminal subtypes, mostly the HER2‐enriched. In a survival analysis, higher relative expression of promC and promB were associated with shorter OS or RFI and DRFI, respectively. Development of resistance to endocrine therapy has been repeatedly associated with activation of other signalling pathways including growth factor receptors and ER‐positive tumours of some molecular subtypes might be primed for this transition. Higher expression of promC has also previously been linked to worse prognosis in tamoxifen‐treated patients and the authors proposed that the presence of different 5′ UTRs could have functional consequences for mRNA half‐life and translational initiation [41]. Several studies have identified mechanisms for translational regulation of ER expression that depend on the 5′ UTR sequence including cap‐independent initiation, upstream open reading frames (uORFs), and G‐quadruplex motifs [42, 43, 44]. We recently published an extensive characterisation of alternative ER isoforms where we showed that these differ in function but that only between 49% and 60% of the best supported alternative protein isoforms would be detected in immunohistochemistry, depending on the antibody used [45]. Likewise, the potential clinical impact of alternative promoter usage on prognostication and therapeutic strategy should be further explored in breast cancer.

One of the regions we included in our methylation analysis (region 6) is not located near an alternative first exon of the ER but appeared interesting because of its accumulation of CpG sites and presence at the start of the intronic antisense lncRNA LOC107986529. This region is mostly unmethylated in our samples, but the highest initial levels of CpG site methylation are seen in the two cell lines that develop long‐term stable resistance, which is also accompanied by increased methylation at specific sites in the region. Expression of the lncRNA was strongly positively correlated with ER expression and methylation of some sites in this region (chr6:151764484 and 151764506) was negatively correlated with ER expression in both breast tumours and our set of cell lines. As previously mentioned, a CpG in region 6 overlaps one of several CTCF binding sites within a 2 kb region and binding of cohesin has been reported to be required for expression of the ER [46]. The lncRNA could potentially act as an enhancer RNA or be involved in tethering of CTCF, which has distinct DNA and RNA binding domains [47]. Interestingly, the sequence of the lncRNA contains two matches to the proposed CTCF RNA consensus motif ‘AGAUNGGA’ [48]. Expression of multiple lncRNAs referred to as *Eleanors* (*ESR1* locus enhancing and activating non‐coding RNAs) within the *ESR1* gene has previously been detected in MCF7 cells subjected to LTED [49]. The authors identified one lncRNA that originated approximately 40 kb upstream of the main promoter region and the first coding exon and showed that knockdown of this lncRNA led to decreased ER expression and cell proliferation and viability. The studied RNA was encoded from the same strand as the ER and would therefore be distinct from the predicted antisense lncRNA that is annotated in the RefSeq database and which we refer to here. However, the RT‐PCR primers and siRNAs that were used all map within the first exon of the antisense RNA and would therefore amplify and target both sequences. Clearly, future research should address the question of (bi‐) directionality and the important role of this DNA region in forming topological domains in chromatin which affect the ER status of the cell.

The results presented here expand upon previous studies by performing methylation analysis for a larger number of CpG sites in more promoter regions with better resolution of methylation levels due to the use of next‐generation sequencing. The inclusion of six different cell lines with matched resistant sublines that differed in phenotype and long‐term stability of resistance allowed us to make comparisons and to study the relationship between methylation and the use of alternative first exons. Two very important questions for future work are whether methylation and expression patterns before drug selection determine the extent and stability of resistance and whether differences in these patterns make some primary tumours more prone to develop resistance so that they could be identified already at primary diagnosis. Increased methylation of enhancer sites for target genes bound by the ER (including the ER itself) has been reported in both cell line models of endocrine resistance and in patients with local recurrence after endocrine therapy [28]. This could shape the cellular response to both ER signalling and therapy. Interestingly, the same study also found differences in enhancer methylation between primary tumours with long‐term progression‐free survival (> 14 years) or local recurrence within 6 years.

## 5 Conclusions

Analysis of six cell line models of fulvestrant resistance showed dynamic changes in methylation of CpG sites and expression of alternative first exons of the ER with striking differences between cell lines with stable or unstable resistance. Many CpG sites overlap TFBSs and methylation at some sites was strongly negatively correlated with expression of specific first exons. Higher relative expression of upstream alternative first exons was also associated with worse prognosis in post‐menopausal women with ER‐positive tumours who received endocrine treatment. Finally, our results point to a possible role for methylation in a region with multiple CTCF sites and an intronically encoded antisense lncRNA in determining ER status.

## Conflict of interest

The authors declare no conflict of interest.

## Author contributions

HP contributed to conceptualisation, supervision, project administration and funding acquisition; JA, KK, GH and HP contributed to methodology; JA, MM, VH and HP contributed to software, formal analysis, investigation and visualisation; JA and HP contributed to validation and writing – original draft; KK, GH, JV‐C and HP contributed to resources; JA, MM, VH, JV‐C and HP contributed to data curation; JA, MM, VH, KK, GH, JV‐C and HP contributed to writing – review & editing.

### Peer review

The peer review history for this article is available at https://www.webofscience.com/api/gateway/wos/peer‐review/10.1002/1878‐0261.13713.

## Supporting information


**Fig. S1.** Bioanalyzer fragment length distribution overlay for bisulfite‐converted DNA from a subset of 10 samples.
**Fig. S2.** Expression of the oestrogen receptor (ER) in parental and derived sublines determined by immunoblotting.
**Fig. S3.** Expression of the oestrogen receptor (ER) in parental and derived CAMA‐1, ZR‐75‐1, EFM‐19, and HCC1428 sublines determined by immunoblotting for two additional replicates.
**Fig. S4.** Expression of the oestrogen receptor (ER) in parental and derived MCF7 and T‐47D sublines determined by immunoblotting for two additional replicates.
**Fig. S5.** Comparison of differences in methylation between sublines for a subset of CpG sites in region 10 and 11 measured by Illumina sequencing and pyrosequencing.
**Fig. S6.** Comparison of differences in methylation between sublines for a subset of CpG sites in region 12 measured by Illumina sequencing and pyrosequencing.
**Fig. S7.** CpG site methylation in publicly available data for resistant MCF7 cells.
**Fig. S8.** Distribution of CpG site methylation patterns per cell line for regions 1–5.
**Fig. S9.** Distribution of CpG site methylation patterns per cell line for regions 6–9.
**Fig. S10.** Distribution of CpG site methylation patterns per cell line for regions 10–12.
**Fig. S11.** Evolutionary conservation of CpG sites.
**Fig. S12.** Methylation at three CpG sites in region 6 was negatively correlated with expression of the oestrogen receptor (ER) in 1095 breast tumours from The Cancer Genome Atlas breast cancer (TCGA BRCA) cohort.
**Fig. S13.** Expression of the intronic LOC107986520 antisense RNA is correlated with expression of the oestrogen receptor (ER) in 3478 breast tumours.
**Fig. S14.** Expression of alternative first exons in 3478 breast tumours.
**Fig. S15.** Expression of alternative first exons as fraction of the total first exon expression in 2968 breast tumours divided by oestrogen receptor (ER) and HER2 receptor status, Nottingham histological grade, and Prediction Analysis of Microarray 50 (PAM50) molecular subtype.
**Table S1.** Sequences of primers and annealing temperatures used in polymerase chain reaction (PCR) amplification of bisulfite‐converted DNA and sequencing primers for pyrosequencing.
**Table S2.** Methylated fraction for 108 CpG sites in 19 sublines, overlapping transcription factor binding sites from the UniBind robust 2021 set including cell type information, phyloP conservation scores, and Spearman rank correlation between methylation beta‐value and ESR1 expression in breast tumours from The Cancer Genome Atlas (TCGA).
**Table S3.** Sequence data statistics from Bismark and the estimated percentage of unconverted Cs based on CHG and CHH sites.
**Table S4.** Spearman rank correlation for first exon expression versus methylated fraction across the 19 sequenced samples with *P*‐values and false discovery rate.
**Table S5.** Univariable and multivariable Cox proportional hazards regression analysis for overall survival (OS), recurrence‐free interval (RFI), and distant recurrence‐free interval (DRFI) in patient groups divided by alternative first exon expression.

## Data Availability

The results shown here are in part based upon data generated by the TCGA Research Network: https://www.cancer.gov/tcga. Due to Swedish law, the patient consent, and the risk that the sequence data contain person‐identifiable information and hereditary mutations, we cannot deposit the SCAN‐B raw sequence data in a repository. The short‐read dataset supporting the conclusions of this article is available in the NCBI Gene Expression Omnibus (GEO) repository, accession number GSE96058, and from the corresponding author upon request. The bisulfite sequencing data has been deposited in the NCBI Gene Expression Omnibus (GEO) repository, accession number GSE251791.
