# Supplementary material for: Dynamic methylation and expression of alternative promoters for oestrogen receptor alpha in cell line models of fulvestrant resistance
Source: Mol Oncol. 2024 Aug 6;19(1):204–24. doi: 10.1002/1878-0261.13713 (PMC11705752; doi:10.1002/1878-0261.13713)
Supplement: Supplementary file 1 — Fig. S1. Bioanalyzer fragment length distribution overlay for bisulfite‐converted DNA from a subset of 10 samples. Fig. S2. Expression of the oestrogen receptor (ER) in parental and derived sublines determined by immunoblotting. Fig. S3. Expression of the oestrogen receptor (ER) in parental and derived CAMA‐1, ZR‐75‐1, EFM‐19, and HCC1428 sublines determined by immunoblotting for two additional replicates. Fig. S4. Expression of the oestrogen receptor (ER) in parental and derived MCF7 and T‐47D sublines determined by immunoblotting for two additional replicates. Fig. S5. Comparison of differences in methylation between sublines for a subset of CpG sites in region 10 and 11 measured by Illumina sequencing and pyrosequencing. Fig. S6. Comparison of differences in methylation between sublines for a subset of CpG sites in region 12 measured by Illumina sequencing and pyrosequencing. Fig. S7. CpG site methylation in publicly available data for resistant MCF7 cells. Fig. S8. Distribution of CpG site methylation patterns per cell line for regions 1–5. Fig. S9. Distribution of CpG site methylation patterns per cell line for regions 6–9. Fig. S10. Distribution of CpG site methylation patterns per cell line for regions 10–12. Fig. S11. Evolutionary conservation of CpG sites. Fig. S12. Methylation at three CpG sites in region 6 was negatively correlated with expression of the oestrogen receptor (ER) in 1095 breast tumours from The Cancer Genome Atlas breast cancer (TCGA BRCA) cohort. Fig. S13. Expression of the intronic LOC107986520 antisense RNA is correlated with expression of the oestrogen receptor (ER) in 3478 breast tumours. Fig. S14. Expression of alternative first exons in 3478 breast tumours. Fig. S15. Expression of alternative first exons as fraction of the total first exon expression in 2968 breast tumours divided by oestrogen receptor (ER) and HER2 receptor status, Nottingham histological grade, and Prediction Analysis of Microarray 50 (PAM50) molecular subtype. Tab [file MOL2-19-204-s001.zip › mol213713-sup-0001-Supinfo.docx]

**Supplementary Figure S1.** Bioanalyzer fragment length distribution overlay for bisulfite-converted DNA from a subset of 10 samples. For comparison, the amplicons used for sequencing were in the 300-450 bp range.

**
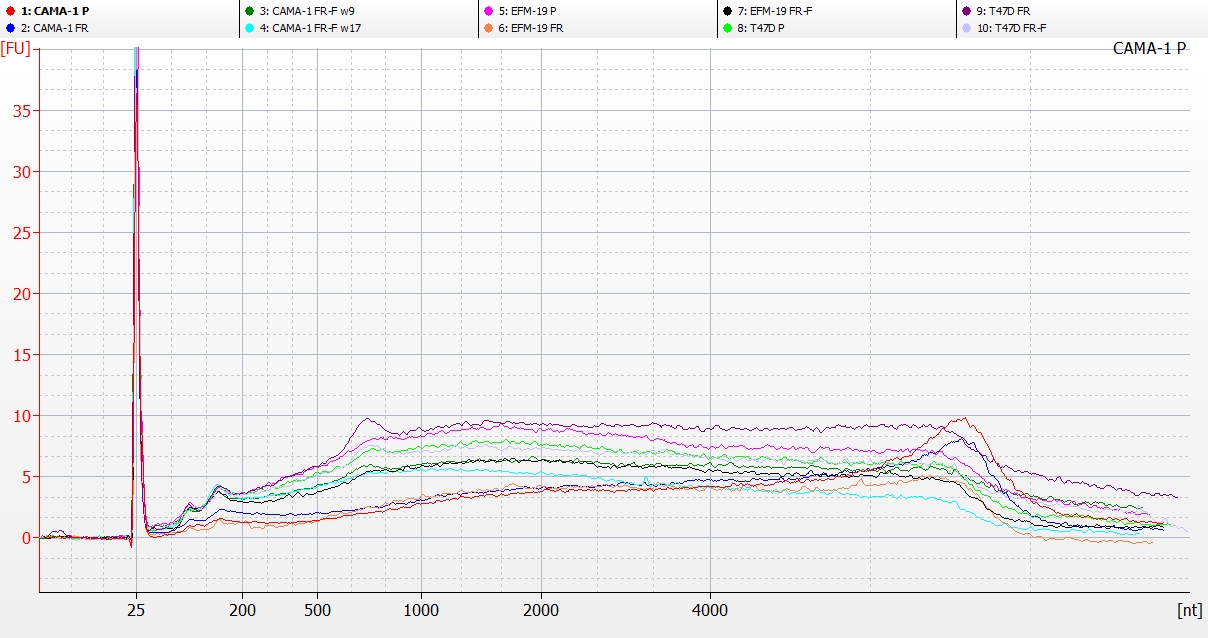
Supplementary Table S1.**  Sequences of primers and annealing temperatures used in polymerase chain reaction (PCR) amplification of bisulfite-converted DNA and sequencing primers for pyrosequencing. 5'-biotinylated versions of the reverse primers were used to prepare PCR templates for pyrosequencing.

| **Name** | **Sequence** | **Annealing temperature in ºC** |
| --- | --- | --- |
| region1_for | AGTTTTAGTGTATAGAATGGAAATTTGT | 52 |
| region1_rev | AAACTTACCTTCTTTAATAACTACAAAA | 52 |
| region2_for | TGGAAAATTTTGTAGGTTAGTTTTGTT | 55 |
| region2_rev | ACAAAACAACAAACAATAACACT | 55 |
| region3_for | AGGGTTGAAGAGTGTGAGAAGT | 60 |
| region3_rev | ACCTTACTTTTACCAAACCAAACC | 60 |
| region4_for | TTTAGTTGAGGTAGAGGGTAATT | 58 |
| region4_rev | TCAAAAACCCATTTCAAAACAAA | 58 |
| region5_for | ATTTGTTTTTTGAATTTTTGGAGTTA | 60 |
| region5_rev | TCCTAATTAAACCCAACCCTTCT | 60 |
| region6_for | GGAGATGTTTTTATTTTGGAGAAGGA | 55 |
| region6_rev | ACTTACAAAATCACATAAAATACTAAAT | 55 |
| region7_for | AGGTGTGGAAGGTAAGGGAA | 60 |
| region7_rev | ACCACTTTCTCTCCAACATTCTCT | 60 |
| region8_for | TGTATAGTGTTTTAGGGTTAGAGA | 55 |
| region8_rev | ACACCAAAAAACCAAACTTTAAA | 55 |
| region9_for | TGGTTGTGTTATATTGTTTTTTGTGA | 60 |
| region9_rev | TCAAAACAAACCTACCCTACT | 60 |
| region10_for | TGTTTTTTATTTGGATATTTGATTTTGT | 55 |
| region10_rev | ACCACAATACTATACTTAATCCCA | 55 |
| region11_for | TTGAGGTTTTGGTAGGTTGT | 60 |
| region11_rev | TCCCAAAAAACAACTTCCCT | 60 |
| region12_for | AGTTATGATGATGATTTTTTGGGA | 55 |
| region12_rev | CCATTAAAATCACAAAATAATCTCCT | 55 |
| region10_pyroseq | AAAATTTGTTAGTTGGATTAGAT |  |
| region11_pyroseq | TAGTTGTTTGTGGTTGGTTG |  |
| region12_pyroseq | TGTTAGTTTAGTGATAAGTTTGT |  |
| ESR1_promA_Forward | CTGTGCTCTTTTTCCAGGTG |  |
| ESR1_promB_Forward | CAGCGACGACAAGTAAAGTG |  |
| ESR1_promC_Forward | GTTCTTGATCCAGCAGGGTG |  |
| ESR1_promD_Forward | CACCTGAGAGAGCCAGTG |  |
| ESR1_promE_Forward | ACCAATCCTTTTGATTGTGAA |  |
| ESR1_promF_Forward | GCATAAGAAGACAGTCTCTGAGTGA |  |
| ESR1_prom_Reverse | AGGGTCATGGTCATGGTC |  |
| ESR1_3UTR_Forward | ACACAGACCCCTTTGCATTC |  |
| ESR1_3UTR_Reverse | TGTAGTGCACAAAAAGCATTG |  |
| ACTB_Forward | GATGACCCAGATCATGTTTGAG |  |
| ACTB_Reverse | CTGGATAGCAACGTACATGG |  |

**
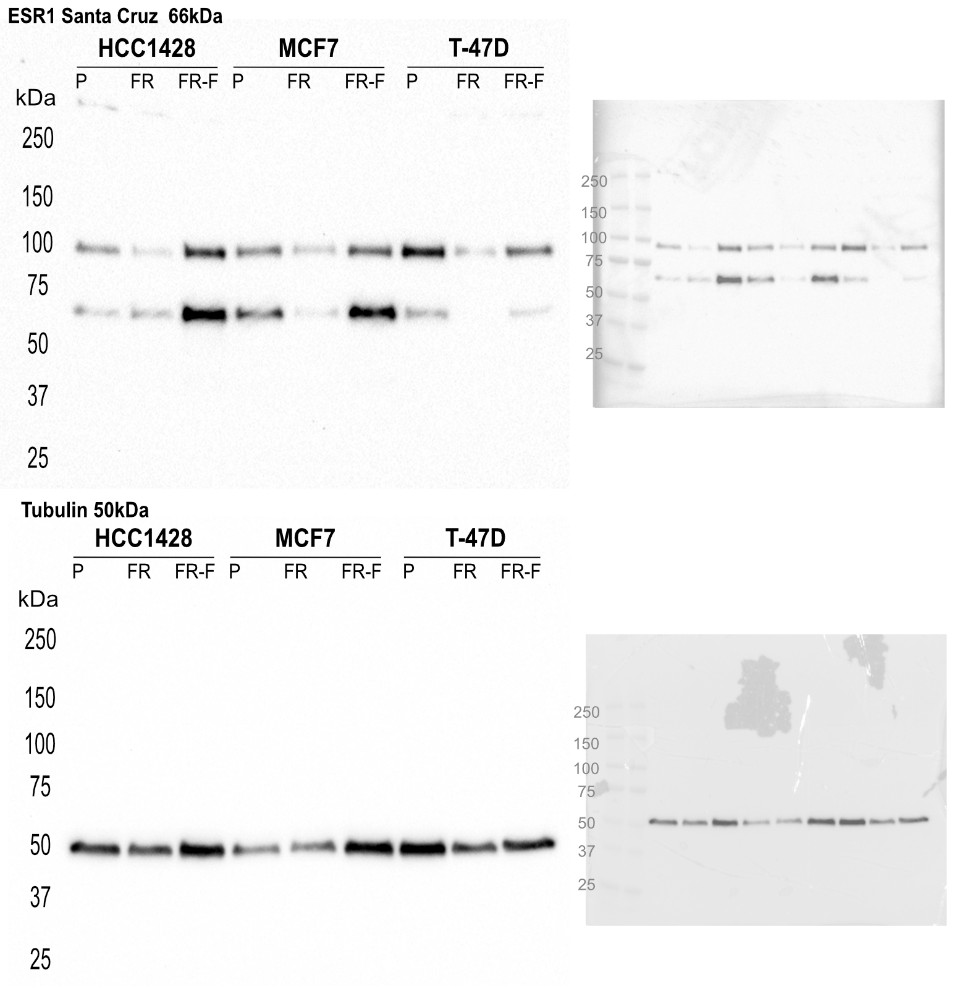

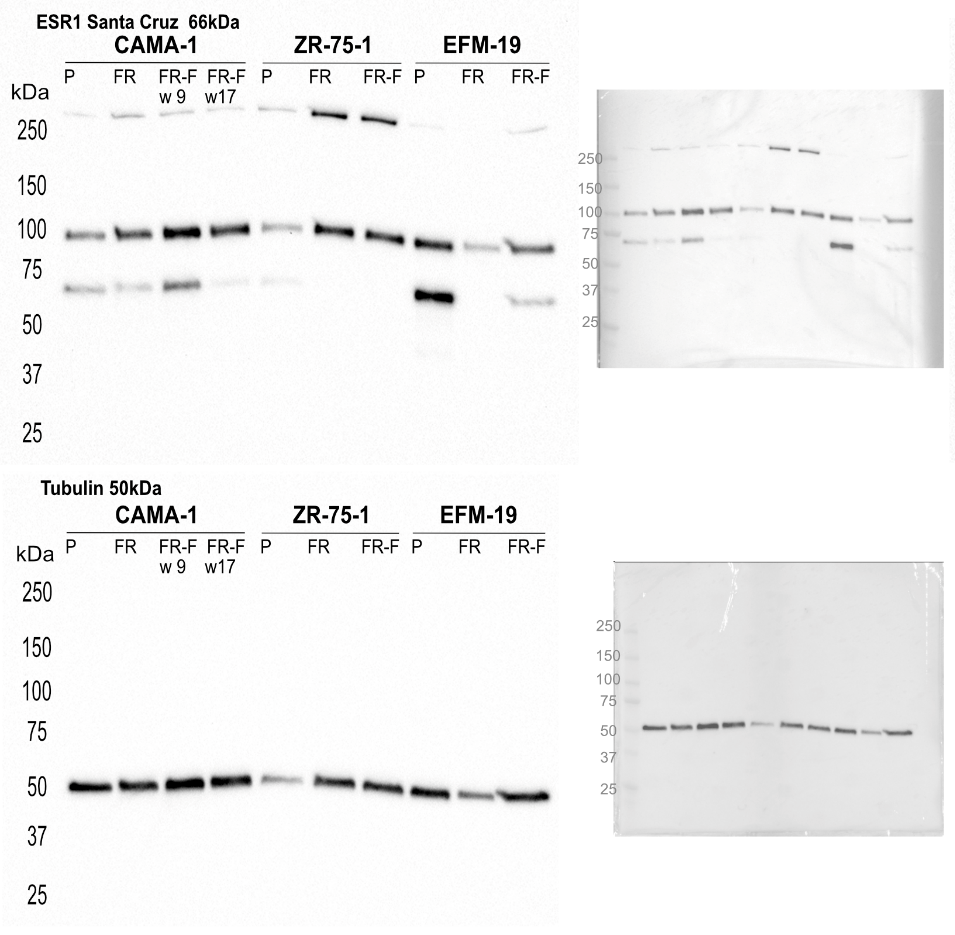
Supplementary Figure S2.** Expression of the oestrogen receptor (ER) in parental and derived sublines determined by immunoblotting. Uncropped blots are shown with and without size marker overlay, n = 1. Membranes were reprobed for tubulin as a loading control. P = parental, FR = fulvestrant-resistant, FR-F = FR cultured without fulvestrant, kDa = kilo Dalton.

**Supplementary Figure S3.** Expression of the oestrogen receptor (ER) in parental and derived CAMA-1, ZR-75-1, EFM-19, and HCC1428 sublines determined by immunoblotting for two additional replicates. Uncropped blots are shown with and without size marker overlay, n = 2. Membranes were reprobed for tubulin as a loading control. P = parental, FR = fulvestrant-resistant, FR-F = FR cultured without fulvestrant, kDa = kilo Dalton.

**Supplementary Figure S4.** Expression of the oestrogen receptor (ER) in parental and derived MCF7 and T-47D sublines determined by immunoblotting for two additional replicates. Uncropped blots are shown with and without size marker overlay, n = 2. Membranes were reprobed for tubulin as a loading control. P = parental, FR = fulvestrant-resistant, FR-F = FR cultured without fulvestrant, kDa = kilo Dalton.

**Supplementary Figure S5.** Comparison of differences in methylation between sublines for a subset of CpG sites in region 10 and 11 measured by Illumina sequencing and pyrosequencing. Methylated fraction for 2 CpG sites in region 10 in HCC1428 and ZR-75-1 measured by (A) Illumina amplicon sequencing and (B) Qiagen pyrosequencing. Methylated fraction for 11 CpG sites in region 11 for ZR-75-1 measured by (C) Illumina amplicon sequencing and (D) Qiagen pyrosequencing. Statistically significant differences between sublines and the parental cell line are indicated for the pyrosequencing data as * *p* < 0.05 and ** *p* < 0.01 (Student’s *t*-test).

**Supplementary Figure S6.** Comparison of differences in methylation between sublines for a subset of CpG sites in region 12 measured by Illumina sequencing and pyrosequencing. Methylated fraction for 9 CpG sites in region 12 in ZR-75-1 measured by (A) Illumina amplicon sequencing and (B) Qiagen pyrosequencing. Methylated fraction for the same CpG sites in HCC1428 measured by (C) Illumina amplicon sequencing and (D) Qiagen pyrosequencing. Statistically significant differences between sublines and the parental cell line (top level) and between the two sublines (lower level where present) are indicated for the pyrosequencing data as * *p* < 0.05, ** *p* < 0.01, and *** p < 0.001 (Student’s *t*-test).**Supplementary Figure S7.** CpG site methylation in publicly available data for resistant MCF7 cells. Comparison between (A) our results and available data for corresponding regions in methylation array datasets for resistant MCF7 cells, (B) GSE69118 where FASR = fulvestrant-resistant, MCF7X = resistant to long-term oestrogen depletion and TAMR = tamoxifen-resistant, as well as (C) GSE240469 and (D) GSE132616 with tamoxifen-resistant cells. Positive values correspond to increased methylation and negative values to decreased methylation compared to parental cells. Regions 1 to 12 are labelled.


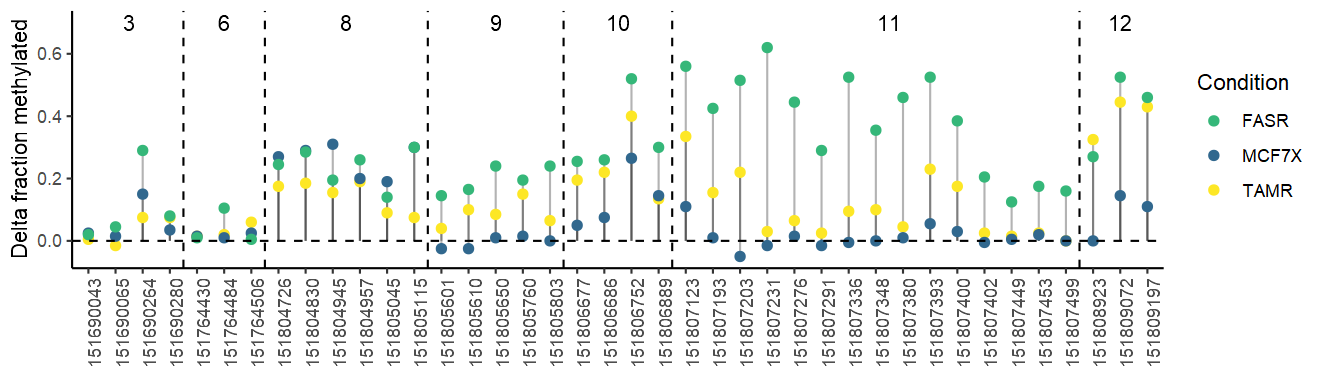

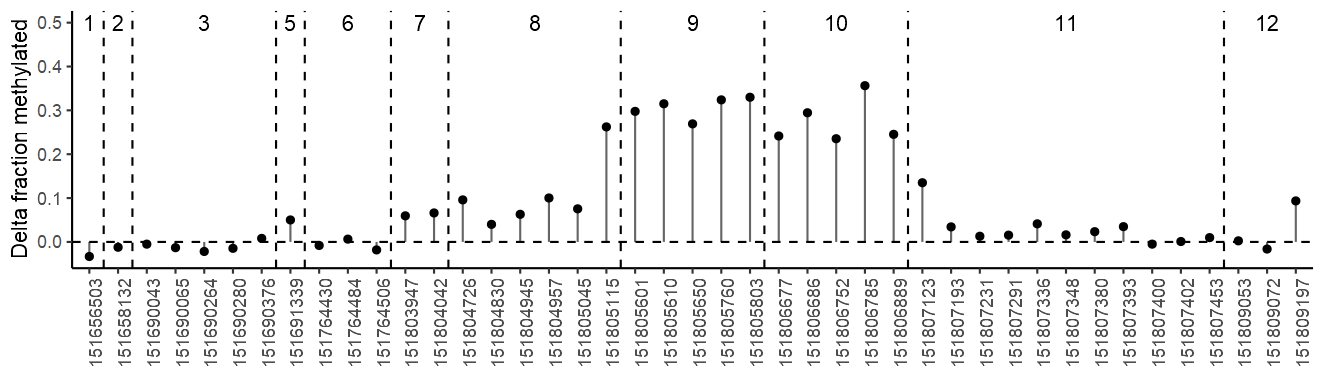

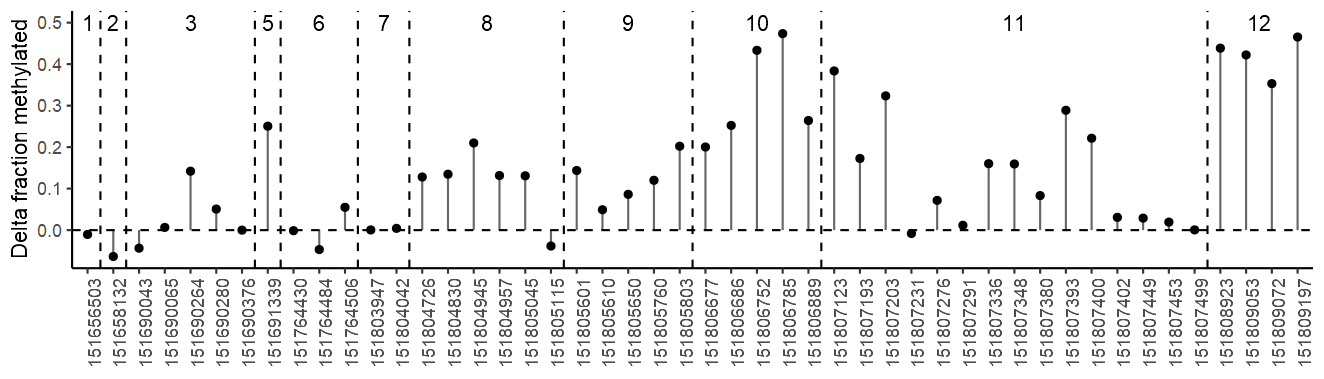


**A**

**B**

**C**

**D**

**Supplementary Figure S8.** Distribution of CpG site methylation patterns per cell line for regions 1-5. Cut-offs for a minimum frequency in at least one of the 19 sequenced samples were used to facilitate visualisation and are indicated below each plot. M = methylated, u = unmethylated, P = parental, FR = fulvestrant-resistant, FR-F = FR cultured without fulvestrant.

**Supplementary Figure S9.** Distribution of CpG site methylation patterns per cell line for regions 6-9. Cut-offs for a minimum frequency in at least one of the 19 sequenced samples were used to facilitate visualisation and are indicated below each plot. M = methylated, u = unmethylated, P = parental, FR = fulvestrant-resistant, FR-F = FR cultured without fulvestrant.

**Supplementary Figure S10.** Distribution of CpG site methylation patterns per cell line for regions 10-12. Cut-offs for a minimum frequency in at least one of the 19 sequenced samples were used to facilitate visualisation and are indicated below each plot. M = methylated, u = unmethylated, P = parental, FR = fulvestrant-resistant, FR-F = FR cultured without fulvestrant.

**Supplementary Figure S11.** Evolutionary conservation of CpG sites. Distribution of phyloP conservation scores for C nucleotides in mammals (A) and vertebrates (B). The solid black lines indicate CpG sites in the 12 analysed regions of the oestrogen receptor (ER) and the dashed lines non-CpG site nucleotides in the same regions. The solid grey lines show the distribution of conservation scores for C nucleotides in CpG sites in 9387 regions for a background set of 3833 genes with similar expression in breast tumours and the dashed grey lines the non-CpG positions. Distribution of the differences between the phyloP scores of C nucleotides in CpG sites and the mean conservation score for each region in mammals (C) and vertebrates (D). A difference (∆phyloP) of 0 means that the C has a conservation score that is identical to the mean for the surrounding region, while a positive ∆phyloP indicates higher conservation and a negative ∆phyloP lower conservation. The solid lines show ∆phyloP scores for CpG sites in the ER regions and the dashed lines CpG sites in the background set.

**Supplementary Figure S12.** Methylation at three CpG sites in region 6 was negatively correlated with expression of the oestrogen receptor (ER) in 1095 breast tumours from The Cancer Genome Atlas breast cancer (TCGA BRCA) cohort (Spearman rank correlation). Methylation is shown as the beta value; the ratio of the methylated signal intensity to the combined intensity for methylated and unmethylated bases. Expression is shown as log2-transformed fragments per kilobase of exon model and million reads (fpkm).

**Supplementary Figure S13.** Expression of the intronic LOC107986520 antisense RNA is correlated with expression of the oestrogen receptor (ER) in 3478 breast tumours (Spearman’s rho = 0.65, *p* < 2.20e-16). Expression is shown as log2-transformed fragments per kilobase of exon model and million reads (fpkm).

**Supplementary Figure S14.** Expression of alternative first exons in 3478 breast tumours. Annotation tracks show Prediction Analysis of Microarray 50 (PAM50) molecular subtype, Nottingham histological grade (NHG) and status for oestrogen receptor (ER), progesterone receptor (PR), the tyrosine kinase receptor ERBB2 (also known as HER2), positive lymph nodes at surgery (LN), and Ki67. ER, PR, HER2, and Ki67 status were defined by immunohistochemistry.

**Supplementary Figure S15.** Expression of alternative first exons as fraction of the total first exon expression in 2968 breast tumours divided by oestrogen receptor (ER) and HER2 receptor status, Nottingham histological grade, and Prediction Analysis of Microarray 50 (PAM50) molecular subtype. A fraction of 1 means that a given first exon is used in all transcripts, while lower values indicate use of alternative first exons. LumA = Luminal A, LumB = Luminal B, Her2 = HER2-enriched, Basal = Basal-like, and Normal = Normal-like subtypes. The Kruskal-Wallis rank sum test was used to calculate *p*-values for multi-group comparisons of PSI scores.

**Supplementary Table S5.** Univariable and multivariable Cox proportional hazards regression analysis for overall survival (OS), recurrence-free interval (RFI), and distant recurrence-free interval (DRFI) in patient groups divided by alternative first exon expression. Only postmenopausal women (defined as age at diagnosis >50 years) with oestrogen receptor (ER) positive tumours who received endocrine therapy were included in the analysis. The number of patients in each group is indicated by “n tumours lo” and “n tumours hi”. The hazard ratios are shown with 95% confidence intervals (CI95) and *p-*values. The multivariable analysis included patient lymph node status (positive or negative), histological grade (1, 2, or 3), tumour size (≤20 mm or >20 mm). * *p* < 0.05, ** *p* < 0.01, *** *p* < 0.001.

| **First exon** | **promE** | **promF** | **promD** | **promC** | **promB** | **promA** |
| --- | --- | --- | --- | --- | --- | --- |
| n tumours lo | 1282 | 1351 | 1231 | 1390 | 1148 | 82 |
| n tumours hi | 147 | 78 | 198 | 39 | 281 | 1347 |
|  |  |  |  |  |  |  |
| *Overall survival (OS) univariable* | | |  |  |  |  |
| HR | 0.73 | 1.50 | 0.82 | 0.43 | 0.95 | 1.70 |
| CI95 low | 0.53 | 0.86 | 0.61 | 0.27 | 0.73 | 1.17 |
| CI95 high | 1.00 | 2.61 | 1.10 | 0.71 | 1.23 | 2.47 |
| *p* | 4.92E-02* | 1.51E-01 | 1.87E-01 | 8.10E-04*** | 6.82E-01 | 5.60E-03** |
|  |  |  |  |  |  |  |
| *Overall survival (OS) multivariable* | | |  |  |  |  |
| *HR* | 0.87 | 1.40 | 0.85 | 0.50 | 0.98 | 1.43 |
| CI95 low | 0.62 | 0.79 | 0.63 | 0.29 | 0.75 | 0.96 |
| CI95 high | 1.21 | 2.50 | 1.15 | 0.85 | 1.28 | 2.13 |
| *p* | 3.95E-01 | 2.53E-01 | 2.86E-01 | 1.14E-02* | 8.71E-01 | 7.59E-02 |
|  |  |  |  |  |  |  |
| *Recurrence-free interval (RFI) univariable* | | | |  |  |  |
| HR | 0.54 | 0.86 | 0.79 | 1.03 | 0.52 | 1.76 |
| *CI95 low* | 0.32 | 0.37 | 0.45 | 0.25 | 0.34 | 0.85 |
| CI95 high | 0.93 | 1.96 | 1.37 | 4.19 | 0.80 | 3.64 |
| *p* | 2.57E-02* | 7.14E-01 | 3.98E-01 | 9.66E-01 | 2.96E-03** | 1.25E-01 |
|  |  |  |  |  |  |  |
| *Recurrence-free interval (RFI) multivariable* | | | |  |  |  |
| *HR* | 0.65 | 0.86 | 0.79 | 1.12 | 0.54 | 1.50 |
| CI95 low | 0.37 | 0.35 | 0.46 | 0.27 | 0.35 | 0.72 |
| *CI95 high* | 1.13 | 2.12 | 1.38 | 4.58 | 0.83 | 3.12 |
| *p* | 1.27E-01 | 7.39E-01 | 4.14E-01 | 8.79E-01 | 5.61E-03** | 2.79E-01 |
|  |  |  |  |  |  |  |
| *Distant recurrence-free interval (DRFI) univariable* | | | | |  |  |
| HR | 0.62 | 0.78 | 0.85 | 0.79 | 0.46 | 2.02 |
| *CI95 low* | 0.33 | 0.31 | 0.45 | 0.19 | 0.29 | 0.93 |
| CI95 high | 1.18 | 1.94 | 1.61 | 3.23 | 0.75 | 4.41 |
| *p* | 1.44E-01 | 5.93E-01 | 6.09E-01 | 7.45E-01 | 1.80E-03** | 7.62E-02 |
|  |  |  |  |  |  |  |
| *Distant recurrence-free interval (DRFI) multivariable* | | | | |  |  |
| HR | 0.69 | 0.78 | 0.88 | 0.88 | 0.49 | 1.65 |
| CI95 low | 0.36 | 0.28 | 0.46 | 0.21 | 0.30 | 0.75 |
| *CI95 high* | 1.31 | 2.15 | 1.69 | 3.65 | 0.79 | 3.63 |
| *p* | 2.57E-01 | 6.28E-01 | 7.10E-01 | 8.64E-01 | 3.98E-03** | 2.16E-01 |
